# PEPstrMOD: structure prediction of peptides containing natural, non-natural and modified residues

**DOI:** 10.1186/s13062-015-0103-4

**Published:** 2015-12-21

**Authors:** Sandeep Singh, Harinder Singh, Abhishek Tuknait, Kumardeep Chaudhary, Balvinder Singh, S. Kumaran, Gajendra P. S. Raghava

**Affiliations:** Bioinformatics Centre, CSIR-Institute of Microbial Technology, Sec 39-A, Chandigarh, 160036 India; CSIR-Institute of Microbial Technology, Sec 39-A, Chandigarh, 160036 India

**Keywords:** Peptide tertiary structure prediction, Peptide structure of non-natural residues, Peptide modeling peptide prediction

## Abstract

**Background:**

In the past, many methods have been developed for peptide tertiary structure prediction but they are limited to peptides having natural amino acids. This study describes a method PEPstrMOD, which is an updated version of PEPstr, developed specifically for predicting the structure of peptides containing natural and non-natural/modified residues.

**Results:**

PEPstrMOD integrates Forcefield_NCAA and Forcefield_PTM force field libraries to handle 147 non-natural residues and 32 types of post-translational modifications respectively by performing molecular dynamics using AMBER. AMBER was also used to handle other modifications like peptide cyclization, use of D-amino acids and capping of terminal residues. In addition, GROMACS was used to implement 210 non-natural side-chains in peptides using SwissSideChain force field library. We evaluated the performance of PEPstrMOD on three datasets generated from Protein Data Bank; i) ModPep dataset contains 501 non-natural peptides, ii) ModPep16, a subset of ModPep, and iii) CyclicPep contains 34 cyclic peptides. We achieved backbone Root Mean Square Deviation between the actual and predicted structure of peptides in the range of 3.81–4.05 Å.

**Conclusions:**

In summary, the method PEPstrMOD has been developed that predicts the structure of modified peptide from the sequence/structure given as input. We validated the PEPstrMOD application using a dataset of peptides having non-natural/modified residues. PEPstrMOD offers unique advantages that allow the users to predict the structures of peptides having i) natural residues, ii) non-naturally modified residues, iii) terminal modifications, iv) post-translational modifications, v) D-amino acids, and also allows extended simulation of predicted peptides. This will help the researchers to have prior structural information of modified peptides to further design the peptides for desired therapeutic property. PEPstrMOD is freely available at http://osddlinux.osdd.net/raghava/pepstrmod/.

**Reviewers:**

This article was reviewed by Prof Michael Gromiha, Dr. Bojan Zagrovic and Dr. Zoltan Gaspari.

**Electronic supplementary material:**

The online version of this article (doi:10.1186/s13062-015-0103-4) contains supplementary material, which is available to authorized users.

## Background

There is a growing interest in the field of peptide therapeutics over the last decade due to numerous adverse effects of chemical drugs [1, 2]. Peptides are becoming popular in the pharmaceutical industry due to their applications in diagnosis, therapeutics and drug delivery with better potency, high specificity, low toxicity, and natural availability [3–7]. Many peptide-based drugs are successfully running in the market and many more are in different phases of clinical trials [8]. Considering the importance of peptides in the therapeutic market, it becomes imperative to know the structural information of a novel peptide prior to its further designing for desired therapeutic properties [9, 10]. It is well established that the function of a peptide depends on its structure, thus it is important to predict the tertiary structure of a peptide from its primary amino acid sequence.

In the past, attempts have been made for the prediction of peptide tertiary structure. In 1999, Ishikawa et al. [11] developed an *ab initio* method (Geocore) for finding the native-like structures within a small ensemble of conformations. However, it was devised as a filtering algorithm instead of a folding algorithm, exploring a large conformational space (~billion conformations) and thereby limiting its use for very small peptides. In 2007, Kaur et al. [12] developed PEPstr algorithm to predict the tertiary structure of small bioactive peptides. They used predicted β-turn and regular secondary structure to build the tertiary structure of a peptide. This approach drastically reduced the time required to build the structure and the method provided a good starting structure by applying the predicted restraints. Nicosia and Stracquadanio (2008) proposed a Generalized Pattern Search Algorithm (Gps) [13] that uses search and poll algorithm to search the global minima. In 2009, Thomas et al. developed PepLook algorithm [14] that is based on Boltzmann-Stochastic technique. Maupetit et al. developed PEP-FOLD [15, 16] algorithm that is based on Hidden Markov Model, greedy algorithm and coarse-grained force fields. A series of 50 greedy simulations are performed for each peptide sequence, generating 50 models. Narzisi et al. [17] proposed a multi-objective evolutionary algorithm (I-PAES) for searching the conformational space based on ECEPP potential energy function. Gps, PepLook and I-PAES, all apply conformational search strategy generating thousands of structures and thereby they may be computationally intensive. PEP-FOLD, however, avoids extensive searching of conformational space by predicting the structural alphabets, which are assembled to provide a starting structure followed by simulations. Recently, Beaufays et al. [18] extended the PepLook algorithm to handle linear and cyclic peptides with non-proteinogenic amino acids. Thevenet et al. [19] updated the PEP-FOLD algorithm to handle the disulfide bonded cyclic peptides. Instead of using distance constraints, they used sOPEP coarse-grained force field. Shen et al. (2014) developed PEP-FOLD2 (improved version of PEP-FOLD) [20] and compared it with PEP-FOLD and Rosetta on a dataset comprising 56 structurally diverse peptides.

Thomas et al. used the Mean Force Potential (MFP) energy values to compare the structures of peptides predicted from PepLook, Robetta and PEPstr with the experimental NMR data and concluded that PepLook and PEPstr models closely resemble the NMR structures [21]. To the best of the authors’ knowledge, at present, only PEPstr and PEP-FOLD methods provide free online service to the worldwide scientific community specifically for the prediction of tertiary structure of peptides from their amino acid sequence. In the past few years, a number of databases have been developed for managing peptides of therapeutic importance that include cell-penetrating, tumor homing, antiparasitic, hemolytic, antihypertensive, anticancer, antimicrobial, quorum-sensing and blood–brain barrier peptides [22–30]. Recently, a meta-database (SATPdb) of therapeutic peptides is developed, which is compiled from twenty-two peptide databases/datasets and can help its users to extract moonlighting peptides with desired function [31]. It has been observed that peptides have poor half-life in circulation and modifications increase their half-life [8, 32, 33]. Different modifications may or may not lead to profound structural changes in the peptide and hence influencing its biological function [34, 35]. In the past, limited attempts have been made to predict the structure of peptides containing non-natural or modified amino acids.

Recently, Gfeller et al. developed SwissSideChain database [36, 37] containing force field library for 210 non-natural residues compatible with Charmm force field in GROMACS and CHARMM software package. Briefly, they generated force field parameters for each non-natural residue either from the analogous natural side-chains or using SwissParam web service [38]. Further, Khoury et al. developed Forcefield_NCAA (FFNCAA) [39], which is a force field library of 147 non-natural amino acids, compatible with ff03 force field in AMBER software package. Starting from initial helical and β-strand conformers, they performed quantum mechanics restrained geometry optimization and further RESP fitting to get the force field parameters for these non-natural residues. Khoury et al. also developed Forcefield_PTM (FFPTM) [40], which is a force field library of 32 frequently occurring post-translational modifications using the same procedure as described above. They also developed web services, FFNCAA and FFPTM, which give the facility of incorporating non-natural amino acid and PTMs respectively to an input PDB file and outputs the modified PDB file to the users. Petrov et al. [41] developed the force field parameters for ~250 different types of PTMs compatible with GROMOS 45a3 and 54a7 force fields in GROMACS. They also developed a web server Vienna-PTM [42], which gives the facility of incorporating any PTM to a PDB file and provide the modified PDB file to the users.

In this study, we have attempted to incorporate special force field libraries for predicting the structure of peptides having non-natural amino acids and different types of PTMs. We employed a logical set of steps that integrates structure prediction software with force field libraries and extended simulations to predict the structures of peptides having non-natural residues and other modifications. We evaluated the performance of PEPstrMOD application on different datasets having modified peptides. We hope that PEPstrMOD will help the scientific community in better understanding of modified peptide structures.

## Methods

### Dataset

In order to evaluate the performance of PEPstrMOD in handling cyclic and modified peptides, we used two datasets. First dataset called “CyclicPep” contains 34 cyclic peptides (obtained from previous studies [18, 19]) having a minimum of one and a maximum of three disulfide bonds in a peptide. Second dataset “ModPep” contains 501 peptides; each peptide has at least one modified residue. In order to generate “ModPep” dataset, first, we extracted 21182 PDB chains having length between 7 and 25. Next, we searched for D-amino acids, non-natural amino acids as specified in FFNCAA and post-translational modifications as specified in FFPTM library and obtained 47, 72 and 692 (total 811) PDB chains. Further, we removed peptides having disulfide bridges and duplicate sequences. Finally, we obtained a dataset of 501 PDB chains called ModPep dataset. We also created a subset “ModPep16” extracted from ModPep dataset comprising 16 peptides, which had regular secondary structure content (helix + strand) ≥60 %. We used DSSP software [43] to assign the secondary structure states of residues in peptides. The eight states (T, S, G, H, I, B, E, −) given by the DSSP software were reduced to three states [44] in which the states ‘G’ and ‘H’ were considered as helix, B and E as strand and the rest of the states as coil.

### Performance measures

We used standard parameters for measuring the performance of our method that is based on Root Mean Square Deviation (RMSD) between predicted and experimentally determined structures. In this study, we computed RMSD for all, only C-alpha and only backbone atoms, which is represented here by RMSD, CA-RMSD and B-RMSD respectively. In order to compute the above parameters, we used Pymol Software [45]. This software first superimposes the predicted and experimentally determined structure of peptides obtained from PDB [46], then it computes different types of RMSD. Hydrogen atoms were removed from the original and the predicted structures using Open Babel [47] before computing RMSD values. If the original structure determined using NMR had multiple models, the best representative structure as defined in the PDB file was used as the original structure. If none of the models was defined as the best representative structure, then, we used the first model as the original structure.

### Defining Rigid Core in NMR structure

Models of an NMR structure may exhibit significant structural diversity. Therefore, it is important to perform an ensemble-level comparison of the original and the predicted structure only within the region of the peptide that is rigid. We, therefore, calculated rigid core (RC) regions in all the NMR peptide structures that had multiple models. We used the same approach as described by Maupetit et al. [15] except that the superposition of NMR models was performed using PROFIT software. Briefly, RC region is defined as residues which exhibit <1.5 angstrom (Å) C-alpha RMS fluctuation.

### PEPstr algorithm

The PEPstr method is explained in detail elsewhere [12], here is a brief overview of PEPstr. This algorithm was developed based on an important observation that β-turns are the major structural constituent of bioactive peptides [48]. PEPstr uses β-turns and secondary structure (Helix, Sheet, Coil) information predicted using BetaTurns and PSIPRED software respectively [44, 49]. The ideal torsion angles (φ, ψ) of the secondary structure (helix: φ = −60, ψ = −40; strand: φ = −120, ψ = 120) and β-turn types [50] are used as restraints. These restraints are used to generate the initial structure using the tleap module of AMBER v6.0. Further, side-chain torsion angles (χ) are assigned to the initial structure using backbone dependent rotamer library [51]. The structure is further energy minimized followed by a short NTP molecular dynamics simulation for 25 picosecond (ps) at 300 K using SANDER module of AMBER with a non-bonded cut-off value of 8 Å. After simulations, a short energy minimization of the structure is performed and the final tertiary structure is predicted.

### PEPstrMOD algorithm

**Fig. 1:**
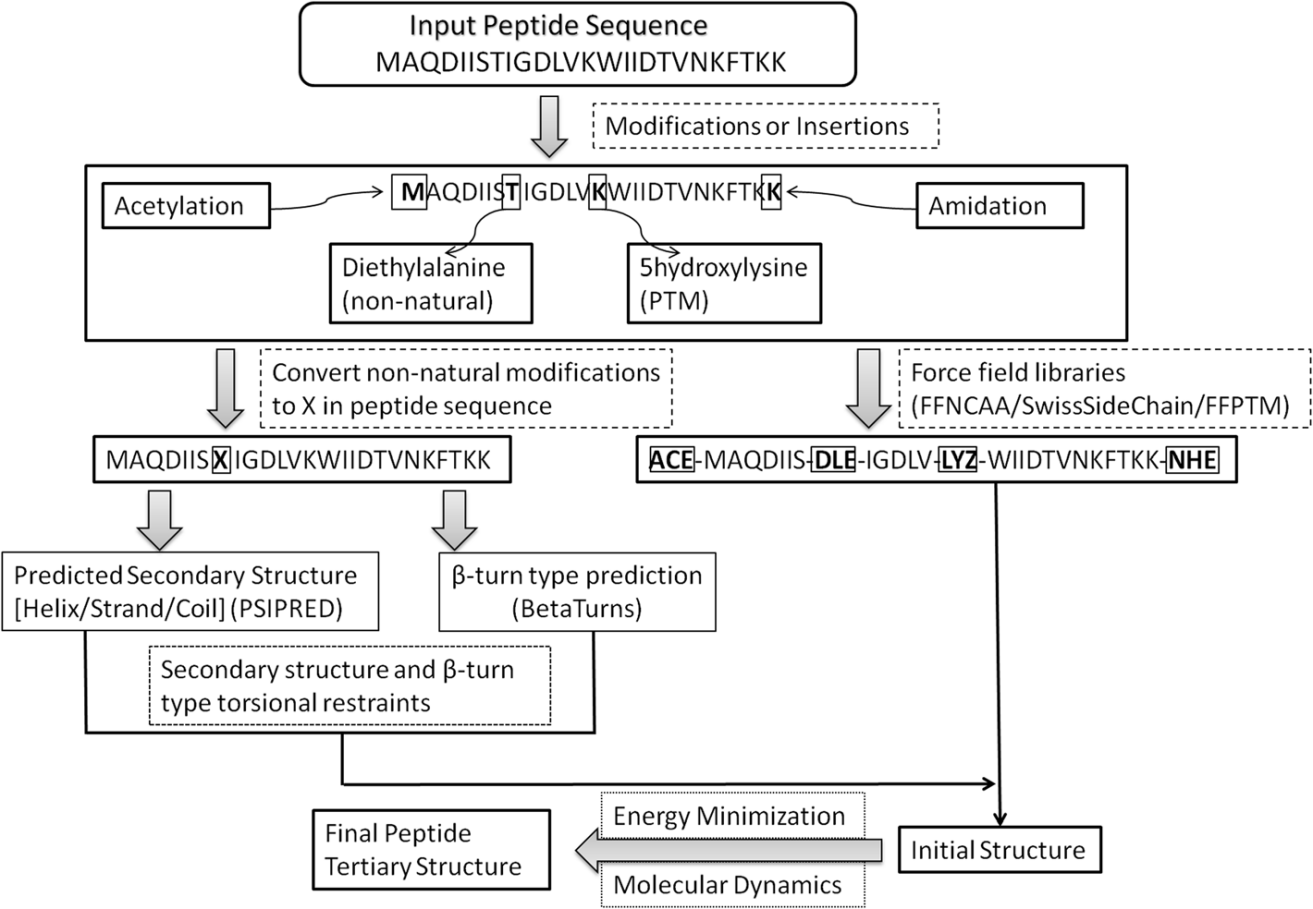
Graphical representation of algorithmic steps of PEPstrMOD showing its working

The original PEPstr method is updated to incorporate most of the generally occurring modifications in the peptides such as terminal modifications, D-amino acids, non-natural amino acids, post-translational modifications, etc. Fig. 1 shows a graphical representation of the algorithmic steps followed by PEPstrMOD. First, a user selects the desired modification or insertion in a given peptide sequence at different positions. Next, the program predicts the secondary structure using PSIPRED and β-turn types using BetaTurns. If the modification incorporated by the user involves any non-natural residue then it is converted to ‘X’ in the peptide sequence before input to PSIPRED and BetaTurns. The ideal torsion angles (φ, ψ) of the secondary structure (helix: φ = −60, ψ = −40; strand: φ = −120, ψ = 120 and β-turn types) are assigned to each residue based upon the predicted secondary structure and β-turn types. The force field libraries (FFNCAA, FFPTM and SwissSideChain) are used to handle the modifications selected by the user in the peptide sequence. After modification in the peptide sequence, an initial structure is generated using tleap module of AMBER v11.0 [52, 53]. The initial structure is subjected to energy minimization and molecular dynamics using either AMBER11 (for FFNCAA/FFPTM) or GROMACS (version 4.6.5) [54, 55] (for SwissSideChain) to generate the final peptide structure.

### Updates and modifications in PEPstrMOD

The updated web server PEPstrMOD (PEPstr with modified residues) can handle natural as well as modified peptides. Apart from incorporation of these modifications, the simulations are performed using AMBER v11.0 instead of old v6.0. The GROMACS (version 4.6.5) molecular dynamics software package is also used for implementing the SwissSideChain force field library. Table 1 shows different modifications, which can be handled using PEPstrMOD and the resources used to handle such modifications. The modifications incorporated in the PEPstrMOD are described below.

**Table 1:** Types of peptides that can be handled with different modifications and the resources used to handle such modifications

Module name	Brief description	Resources used
Natural peptides	Prediction of peptides having natural residue.	PEPstr algorithm using AMBER11.
D-amino acids	Incorporation of D amino acids in a peptide.	Using inbuilt ‘flip’ command in AMBER11
Terminal modifications	Acetylation at N-terminus and/or amidation/N-methylamide group at C-terminus.	Using existing force field parameters in AMBER11.
Peptide cyclization	N-C cyclization of peptides or peptides having disulfide bridges.	Using inbuilt ‘bond’ command in AMBER11.
Non-natural modification	Incorporation of any of the 147 non-natural residues. (e.g. Homoserine, N-alkylated residues, β-substituted residues etc.).	FFNCAA library comprising 147 non-natural residues compatible with AMBER11.
	Incorporation of any of the 210 non-natural residues. (e.g. Ornithine, Norvaline, Halogenated residues etc.).	SwissSideChain library comprising 210 non-natural residues compatible with GROMACS.
PTMs of residue	Peptides with any of the 32 diverse PTMs. (e.g. phosphorylation, palmitoylation, hydroxylation etc.).	FFPTM library compatible with AMBER11.
Advance modification	Combination of all the above six modules to provide facility to incorporate multiple modifications in one step.	All the resources used in the above modules.
Structure simulations	Facility to provide extended simulations.	All the resources used in the above modules.

#### Terminal modifications

The most common terminal modifications are acetylation at N-terminus and amidation at C-terminus. These terminal modifications protect the peptides from degradation by exopeptidases. PEPstrMOD uses inbuilt functions present in the AMBER11 package to incorporate acetylation at N-terminus and amidation/N-methylamide modification at C-terminus and predicts the peptide conformation.

#### Stereo-chemical modifications

Replacement of L-amino acid by stereo-chemically modified D-amino acid helps in providing stability to the peptide from enzymatic breakdown. It can also provide insights into the stereo-structural requirements of certain secondary structures (promoting reverse β-turn conformations), which play an important role towards the bioactivity of the peptide [56]. PEPstrMOD uses “flip” command of AMBER11 to replace the L-amino acid with the D-amino acid.

#### Peptide cyclization

In order to restrict the conformational flexibility and to provide additional stability, peptides are cyclized either terminally (N-C cyclization) or by covalent side-chain cyclization using disulfide bridge between thiol group of two cysteine residues. To achieve N-C cyclization, PEPstrMOD uses inbuilt “bond” command of AMBER11 to build a bond linking the N-terminal amino group with the C-terminal carboxyl group. Similarly, for disulfide linkage, the SG atoms of the thiol group of two cysteine amino acids are covalently linked (using “bond” command in AMBER11) to achieve side-chain-to-side-chain cyclization.

#### Non-Natural modifications

Common non-natural modifications include, but are not limited to, N-Alkylation, C^α^-tetrasubstituted α-amino acids (e.g. Aib [α-amino butyric acid]), β-substituted amino acids, etc. These non-natural amino acids cannot be directly handled by standard force field libraries implemented in AMBER and GROMACS. PEPstrMOD uses special force field libraries FFNCAA and SwissSideChain, to incorporate these non-natural amino acids in the peptide followed by energy minimization and molecular dynamics to generate the tertiary structure of the peptide having these modifications. Briefly, FFNCAA library contains parameters for 147 non-natural/noncanonical amino acids, which are compatible with AMBER software package, and SwissSideChain represents 210 non-natural amino acids with both L- and D-configurations, which are compatible with GROMACS software package. Together, these libraries represent diverse modifications, which include alkylation, β-amino acids, methoxylation, halogenation, sulfones, etc. The list of non-natural amino acids from FFNCAA and SwissSideChain force field libraries is available in the Additional file 1: Table S1.

#### Post Translational Modifications (PTMs)

Many functional aspects of proteins/peptides are regulated through PTMs. PTMs (e.g. phosphorylation, hydroxylation, etc.) regulate a variety of functions like regulation of gene expression, signaling cascades, etc. With the availability of special force field library for PTM (FFPTM), developed using quantum-chemical level, the integration and use of these force fields in PEPstrMOD makes it feasible to study the effect of PTMs on the structure of peptides. Briefly, FFPTM library consists of parameters for 32 common PTMs (e.g. phosphorylation, acetylation, hydroxylation, palmitoylation, farnesylation, etc.), which are compatible with AMBER software package. The list of PTMs from FFPTM force field library is available in the Additional file 1: Table S2.

#### Molecular dynamics details

Due to the compatibility issues of the available force fields, we used two molecular dynamics software packages, AMBER version 11 (compatible with FFNCAA and FFPTM libraries) and GROMACS version 4.6.5 (compatible with SwissSideChain library). A non-bonded cut-off value of 10 Å was used and minimization was performed for 2000 steps (1000 steps with steepest descent and rest of the steps with conjugate gradient algorithm). Next, the system was heated for 50 ps (ps) using NVT ensemble (at 300 K) and then equilibrated for 50 ps at NPT ensemble (at pressure of 1 bar). Finally, production molecular dynamics was performed at 300 K temperature and 1 bar pressure with a time step of 1 femtosecond. For performing simulation in water, TIP3P [57] and SPC [58] water models were used in AMBER and GROMACS respectively and for simulations in hydrophobic environment, methane box was used. The simulation time (50/100 ps) can be selected by the user. Simulation can be performed either in water, hydrophobic or vacuum environments. In vacuum environment, the system was heated for 50 ps (at 300 K) followed by production molecular dynamics (at temperature of 300 K) with no periodic boundary conditions. All the parameters corresponding to molecular dynamics simulations in AMBER and GROMACS used in PEPstrMOD are provided in Additional file 1: Table S3 and S4.

## Results and discussion

PEPstrMOD implements the original PEPstr algorithm and incorporates modified residues using force field libraries. The PEPstr algorithm has already been benchmarked on a set of 42 bioactive peptides [12]. Other methods, like PEP-FOLD, also compared and evaluated the performance of PEPstr algorithm with their methods [15, 16]. Here, we evaluated the application of PEPstrMOD in handling the peptides having modified residues. Table 2 shows the types of modifications implemented in the PEPstrMOD method along with the availability of these modifications in other peptide tertiary structure prediction methods.

**Table 2:** Types of modifications available in PEPstrMOD and availability of these modifications in other methods

Prediction methods	Natural peptides structure prediction	Peptide modifications					
		N and C terminal	L/D	Cyclization	Non-natural residues covered	PTM	Availability
PEPstrMOD	Y	Y	Y	Y	210^a^ +147^b^	32^c^	Web-service
PEP-FOLD (2012)	Y	N	N	Y	N	N	Web-service
PepLook −2011	Y	N	Y	Y	~19	N	N
Narzisi et al. (2010)	Y	N	N	N	N	N	N
PEP-FOLD (2009)	Y	N	N	N	N	N	Web-service
PepLook −2009	Y	N	N	N	N	N	N
Gps −2008	Y	N	N	N	N	N	N
PEPstr −2007	Y	N	N	N	N	N	Web-service
Geocore −1999	Y	N	N	N	N	N	N

Y: Available

N: Not-Available

^a^list of non-natural residues from SwissSideChain library

^b^list of non-natural residues from FFNCAA library

^c^list of non-natural residues from FFPTM library

### Performance of PEPstrMOD on CyclicPep dataset

We cyclized the structure of peptides using PEPstrMOD and computed its performance to determine its utility in peptide cyclization. In order to provide a direct comparison with the existing method PEP-FOLD, we used PEP-FOLD server for computing structure of peptides. We predicted the structure of each peptide in CyclicPep dataset using PEP-FOLD server without giving disulfide bond constraints. Next, we used PEPstrMOD for cyclization of these predicted peptide structures using “Structure Modification” sub-module of “Peptide Cyclization” module. We represent these cyclic peptide structures by set PEPstrMOD^pc^. We also predicted the cyclic structure of peptides in CyclicPep dataset using PEP-FOLD with disulfide bond constraints given to the PEP-FOLD. We represent these cyclic peptide structures computed directly from PEP-FOLD by set PEP-FOLD^c^. Finally, we evaluated the performance of peptide structures in both PEPstrMOD^pc^ and PEP-FOLD^c^. As shown in Table 3, PEP-FOLD^c^ achieved an average CA-RMSD of 4.16 Å while PEPstrMOD^pc^ achieved 4.06 Å respectively. These results indicate that PEPstrMOD has the ability to cyclize the peptide structure with reasonable precision, comparable to existing server PEP-FOLD. Out of 34 peptides, PEPstrMOD performed better than PEP-FOLD in terms of B-RMSD for almost half of the cases and vice versa (Additional file 2: Table S5). Considering the difference in B-RMSD of >1 Å, PEPstrMOD performed better than PEP-FOLD in 10 cases while PEP-FOLD was better in 7 cases (Additional file 2: Table S5). In the rigid core (RC) region, the same trend was observed i.e. the overall performance of PEPstrMOD and PEP-FOLD was comparable with average CA-RMSD of 3.69 Å and 3.74 Å respectively (Additional file 2: Table S5).

**Table 3:** The performance of different methods on peptides in CyclicPep dataset. All models were subjected to 100 ps molecular dynamics simulations in vacuum environment

		*ab initio* model		PEP-FOLD^c^		PEPstrMOD^pc^	
PDB ID	L^a^	CA-RMSD	B-RMSD	CA-RMSD	B-RMSD	CA-RMSD	B-RMSD
1n0c	10	2.98	2.31	2.30	1.83	0.99	0.83
1n0a	11	3.61	3.92	0.50	0.41	1.09	1.21
1etl	12	2.07	2.10	3.14	2.47	2.51	2.46
1im1	12	3.76	3.86	1.85	1.90	2.39	2.44
1gnb	13	3.45	3.06	5.18	5.13	3.84	3.86
1hje	13	3.56	3.60	3.64	3.64	3.85	2.47
1im7	13	4.33	3.51	4.07	3.74	3.73	3.55
1xgb	13	3.91	3.44	3.59	3.59	2.51	2.62
2i28	13	3.29	3.23	1.16	0.79	4.66	4.24
1b45	14	2.93	1.97	3.74	2.17	3.98	2.36
1jbl	14	4.26	3.36	2.10	2.05	4.01	3.13
1r8t	15	3.22	2.97	3.84	3.70	2.33	2.08
1kwd	16	2.24	2.58	2.76	2.18	4.58	4.53
1mii	16	4.35	4.36	0.86	0.88	2.69	2.46
2efz	16	5.37	5.27	4.54	3.75	3.40	3.31
1nim	17	4.35	4.40	3.68	3.53	5.54	5.37
1ien	19	5.01	4.70	2.54	2.44	2.49	3.46
1x7k	19	4.79	4.73	5.03	4.92	4.43	4.28
1kcn	21	4.49	4.41	6.17	5.74	6.58	6.24
1rpc	21	6.34	6.36	5.86	5.85	6.03	5.96
1ter	21	7.39	7.26	5.57	5.44	2.56	2.64
1v6r	21	7.08	7.03	5.91	5.88	5.69	5.85
1 hp9	22	5.35	5.38	2.76	2.70	2.40	2.03
2ajw	22	4.88	4.76	0.99	0.83	2.81	2.64
1oig	24	6.82	6.69	5.56	5.59	6.16	6.14
1orx	24	7.61	7.41	6.97	6.81	5.85	5.85
1sp7	24	6.40	6.46	6.47	5.60	5.98	3.80
2oq9	24	6.28	5.76	9.73	9.56	6.36	5.90
1wqc	26	6.57	5.03	1.19	1.12	2.10	2.07
1v5a	28	6.28	6.17	5.06	4.84	3.93	3.76
1wm8	28	7.78	7.87	6.87	7.07	7.89	7.69
2it7	28	7.05	7.06	5.59	5.43	4.40	4.25
2nx7	28	5.65	5.68	6.40	6.31	4.61	4.32
1mmc	30	7.76	6.01	5.97	5.61	5.80	5.70
Average		5.04	4.78	4.16	3.93	4.06	3.81

PEP-FOLD^c^: PEP-FOLD predicted peptide structure using cyclic (disulfide bond) constraints

PEPstrMOD^pc^: PEPstrMOD predicted peptide structure using cyclic (disulfide bond) constrains

*CA-RMSD* C-alpha Root Mean Square Deviation, *B-RMSD* Backbone Root Mean Square Deviation

^a^Length of the peptide

We also extended the duration of MD simulations from 100 ps to 1 nanosecond (ns) and compared the performance of PEPstrMOD at both of these time steps. We did not observe any improvement in the results by extending the MD runs (Additional file 2: Table S5). We also repeated the above experiments while performing the MD simulations in hydrophilic environment instead of vacuum (Additional file 2: Table S6). PEPstrMOD achieved an average CA-RMSD of 3.97 Å in hydrophilic environment, which is slightly better as compared to the performance in vacuum. The performance was further improved from 3.97 Å to 3.82 Å (CA-RMSD) by extending the duration of MD runs from 100 ps to 1 ns in hydrophilic environment (Additional file 2: Table S6).

Due to the unavailability of PepLook service, we were unable to perform the same experiments (like PEPstrMOD^pc^) using initial structure predicted from PepLook; however, we report the result of PepLook, PEP-FOLD and PEPstrMOD on a subset (28 common peptides) of this dataset (Additional file 2: Table S7). The results on 28 cyclic peptides follow the same trend with average CA-RMSD values of 3.91 Å and 3.95 Å by PEPstrMOD^pc^ and PEP-FOLD^c^ respectively. A difference of 0.32 Å is observed in the average B-RMSD values of PEP-FOLD (without constraints) and PEP-FOLD^#^ (results as reported in PepLook Study). This may be because the PEP-FOLD server was updated after the PepLook study. (Additional file 2: Table S7).

### Performance of *ab initio* models on CyclicPep dataset

**Fig. 2:**
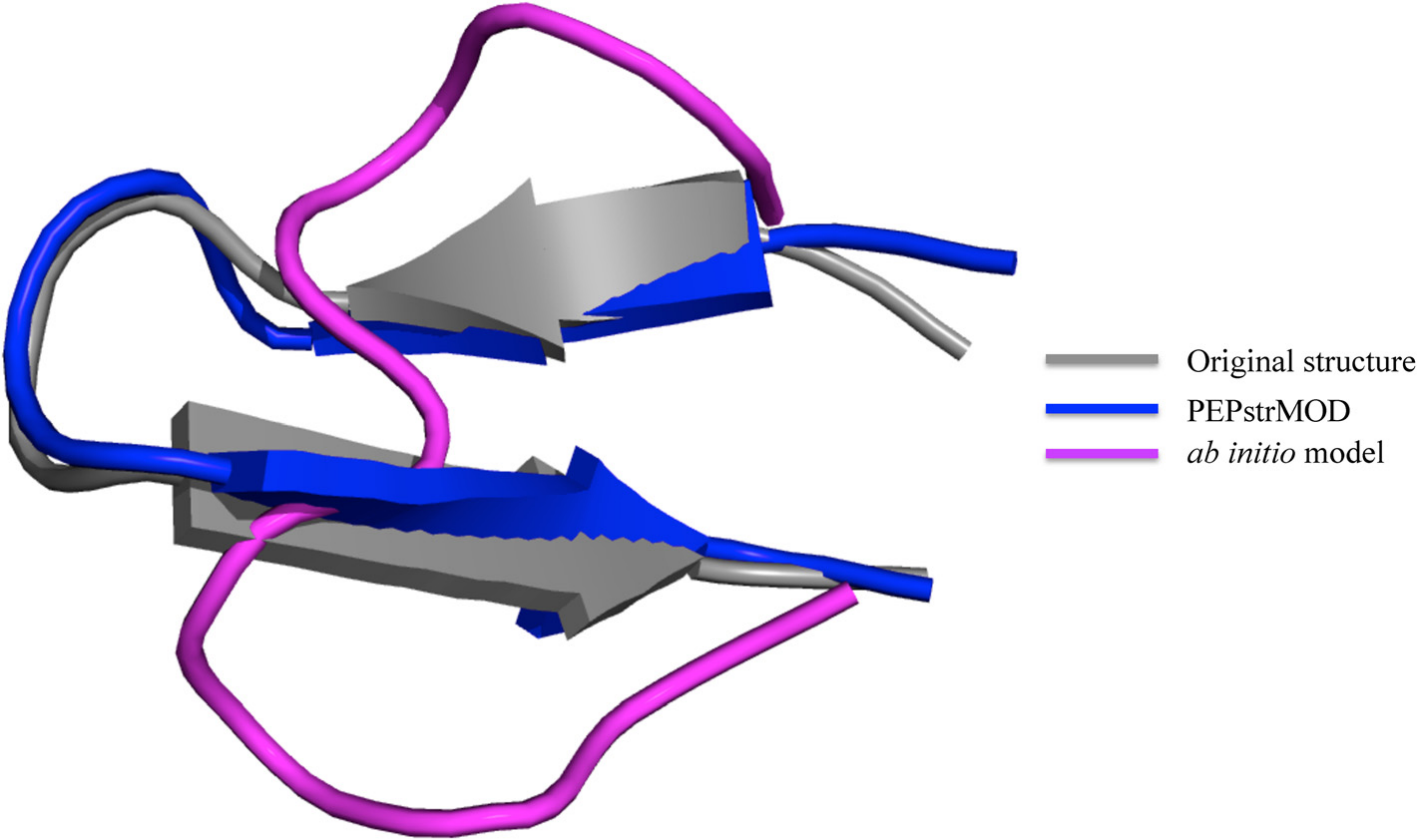
A case study of the comparison of PEPstrMOD and *ab initio* model of a short cyclic peptide (1n0c)

One might argue that the moment one connects the correct cysteine residues in peptides via disulfide bridges, the near-correct topology is immediately attained, especially for very short peptides. In other words, we might not need a sophisticated prediction method for cyclic peptides as merely connecting the correct cysteine residues in peptides followed by geometry optimization, may provide a near-correct topology/structure. To address this point, we performed an additional experiment and predicted the *ab initio* models using an extended conformation with disulfide bond constraints and subjected to 100 ps MD simulation and extended up to 1 ns. We compared the performance of *ab initio* models with PEPstrMOD. PEPstrMOD achieved an average CA-RMSD of 4.06 Å while *ab initio* model achieved 5.04 Å (Table 3). We observed no improvement in the results by extending MD simulation up to 1 ns. Even in the case of very short peptides, PEPstrMOD performed better than *ab initio* models. Figure 2 shows an example of a short cyclic peptide (10 residues) where PEPstrMOD approached close to the native structure while the *ab initio* model performed poor. In the RC region, PEPstrMOD achieved an average CA-RMSD of 3.69 Å while *ab initio* models achieved 4.61 Å. The same trend was observed when the MD simulations were performed in hydrophilic environment (Additional file 2: Table S6).

### Performance of PEPstrMOD on ModPep dataset

In order to demonstrate the application of our algorithm, we implemented it on peptide structures in the ModPep dataset. This dataset contains 501 peptides whose structures are available in the PDB and each peptide has at least one modified residue. We predicted the structure of each peptide using our algorithm and generated an initial structure (starting structure). In the next step, we performed energy minimization on the initial structure to get the minimized structure. Next, we compared our predicted structures with the actual structures in order to compute the performance of our algorithm in terms of RMSD values.

The initial structure obtained using PEPstrMOD achieved an average CA-RMSD and B-RMSD of 4.21 Å and 3.96 Å respectively. After energy minimization, the performance improved to average CA-RMSD and B-RMSD of 4.12 Å and 3.85 Å respectively. In the RC region, the initial and minimized structure achieved an average B-RMSD of 3.76 Å and 3.64 Å respectively (Additional file 2: Table S8). We did not perform the MD simulations on all of the 501 peptides but only on a subset of 501 peptides, which had regular secondary structure content ≥60 % (ModPep16 dataset) and its results are described in the following sections.

### Performance of PEPstrMOD on ModPep16 dataset

ModPep16 dataset contains 16 peptides and is a subset of ModPep dataset. These peptides have regular secondary structure content (helix + strand) ≥60 %. We compared the initial structure, minimized structure and the structure generated after performing 100 ps MD simulations with the actual structures to compute the performance of PEPstrMOD on these peptides. The initial structure generated by PEPstrMOD achieved an average CA-RMSD of 4.83 Å while the performance of minimized structure and structure after 100 ps MD simulations improved to 4.78 Å and 4.31 Å respectively (Additional file 2: Table S9-S11, Table 4). We observed no improvement in the results by extending MD simulation from 100 ps to 1 ns (Additional file 2: Table S9−S12). Performing MD simulations in hydrophilic environment also produced similar results with an average CA-RMSD of 4.35 Å as compared to 4.31 Å in vacuum (Additional file 2: Table S13−S15).

**Table 4:** The performance of different models on 16 peptides in ModPep16 dataset. All models were subjected to 100 ps molecular dynamics simulations in vacuum environment

		*ab initio* model		PEPstrMOD		DSSP model	
PDB ID	L^a^	CA-RMSD	B-RMSD	CA-RMSD	B-RMSD	CA-RMSD	B-RMSD
1fevA	15	5.25	5.12	2.20	2.57	1.82	1.78
1rbdS	15	5.16	5.07	4.86	4.93	5.64	5.08
1tkqB	15	6.46	5.51	6.11	5.95	5.65	5.46
1z3lS	15	5.93	5.45	4.28	3.33	4.78	4.65
1z3mS	15	5.57	5.49	4.81	4.94	3.67	1.73
1z3pS	15	3.39	3.61	4.16	3.90	6.72	6.62
2ap8A	20	4.72	4.78	1.37	0.90	1.17	0.91
2dprA	21	5.60	5.41	1.65	1.52	0.92	0.90
2fx8P	12	4.17	4.15	4.76	4.77	0.88	0.78
2k7lB	19	5.99	6.04	6.60	6.44	0.90	0.98
2rlnS	15	4.16	4.00	6.07	4.69	6.69	2.55
3cmhA	15	5.21	5.26	4.83	4.75	4.59	4.41
3kmzC	19	5.32	4.83	1.69	1.24	2.65	2.77
3zs2D	25	7.23	7.33	5.23	4.85	5.86	5.70
4lkaB	12	4.65	3.93	4.39	4.17	3.25	2.73
6cmhA	21	8.81	8.71	5.96	5.80	5.56	3.87
Average		5.48	5.29	4.31	4.05	3.80	3.18

*CA-RMSD* C-alpha Root Mean Square Deviation, *B-RMSD* Backbone Root Mean Square Deviation

^a^Length of the peptide

### Performance of *ab initio* and DSSP models on ModPep16 dataset

PEPstrMOD is based on predicted secondary structure, and therefore, its performance depends on the method used for predicting the secondary structure of peptides. In order to understand the advantages and limitations of secondary structure prediction method used in PEPstrMOD, we developed two additional models. Firstly, we developed *ab initio* model in which no secondary structure information was used. Secondly, we developed the model based on the observed or actual secondary structure (assigned using DSSP) called DSSP model. The performance of these models is shown in Additional file 2: Table S9−S11 and Table 4. We achieved an average CA-RMSD of 10.94 Å for initial structure generated by *ab initio* models, which improved slightly with energy minimization step (10.77 Å). As shown in Table 4, *ab initio* models achieved an average CA-RMSD 5.48 Å after MD simulations of 100 ps. The performance of PEPstrMOD (4.31 Å) is far better than *ab initio* models that show the importance of predicted secondary structure in model building (Table 4 and Additional file 2: Table S9−S11).

The performance of initial structure of DSSP model achieved an average CA-RMSD of 2.78 Å, which reduced with energy minimization step (2.90 Å) and finally reached to 3.80 Å after performing 100 ps MD simulation (Additional file 2: Table S9−S11). This is due to the fact that the initial structure of DSSP model already achieves a very good starting point due to original structural restraints and there is no scope of further improvement of this structure by MD runs. These results clearly indicate the limitations of secondary structure prediction methods used in our method PEPstrMOD. Thus there is a need to develop better methods for predicting the secondary structure of peptides as presently we are using secondary structure prediction methods developed for proteins. In summary, the performance of PEPstrMOD is better than *ab initio* model but poorer than DSSP model.

### Performance of PEPstrMOD on peptides with different length distributions

We divided CyclicPep and ModPep datasets into three subsets based on the length distribution of the peptides. We ensured that each subset contains sufficient number of peptides. On both the datasets, the performance of PEPstrMOD decreased with the increase in length of the peptide. On CyclicPep dataset, the average B-RMSD achieved by PEPstrMOD was 2.6 Å, 4.06 Å and 4.95 Å in the length range of 10–15, 16–22 and 23–30 amino acids respectively (Table 5). Analyzing peptides with poor performance (>5 Å B-RMSD), a general trend is that both the methods viz. PEPstrMOD and PEP-FOLD tend to perform poor when the length of the peptide is >20 residues (8 cases with PEPstrMOD and 12 cases with PEP-FOLD out of total 16 cases) (Table S5). A similar trend was observed on ModPep dataset with average B-RMSD of 2.91 Å, 4.75 Å and 5.44 Å in the length range of 7–10, 11–15 and 16–25 amino acids respectively (Table 5).

**Table 5:** The performance of PEPstrMOD on peptides in CyclicPep and ModPep datasets having length in different range. All models were subjected to 100 ps molecular dynamics simulations in vacuum environment

CyclicPep dataset					
Length range	Peptides^a^	CA-RMSD	Percent^b^	B-RMSD	Percent^b^
10–15	12	2.99	100	2.60	100
16–22	12	4.10	66.7	4.06	66.7
23–30	10	5.31	40	4.95	50
All (10–30)	34	4.06	70.6	3.81	73.5
ModPep dataset					
Length range	Peptides^a^	CA-RMSD	Percent^b^	B-RMSD	Percent^b^
7–10	279	3.16	88.9	2.91	91.4
11–15	134	4.96	58.2	4.75	61.9
16–25	88	5.88	51.1	5.44	55.7
All (7–25)	501	4.12	74.1	3.85	77.2

*CA-RMSD* C-alpha Root Mean Square Deviation, *B-RMSD* Backbone Root Mean Square Deviation

^a^Number of Peptides

^b^Percent of peptides with B-RMSD <5 Å

### Justification of Force Fields

The force field parameters implemented in PEPstrMOD are adopted from FFNCAA/FFPTM/SwissSideChain and are evaluated and validated in their corresponding research work [37, 39, 40]. Briefly, the force field parameters derived in FFNCAA and SwissSideChain were validated by predicting the binding free energy of a set of analogs and correlating it with experimental values. The force field parameters derived in FFPTM were validated by comparing the RMSD differences between the original natural PDB structure plus desired modified residue (followed by molecular dynamics) and the modified PDB structure. They observed the final state of the structure to be same with comparable side-chain distributions. Moreover, in our study, we observed that using the force fields of modified residues, PEPstrMOD is able to approach towards the native structure (Additional file 2) which further validates the application of this study.

### Web Implementation

**Fig. 3:**
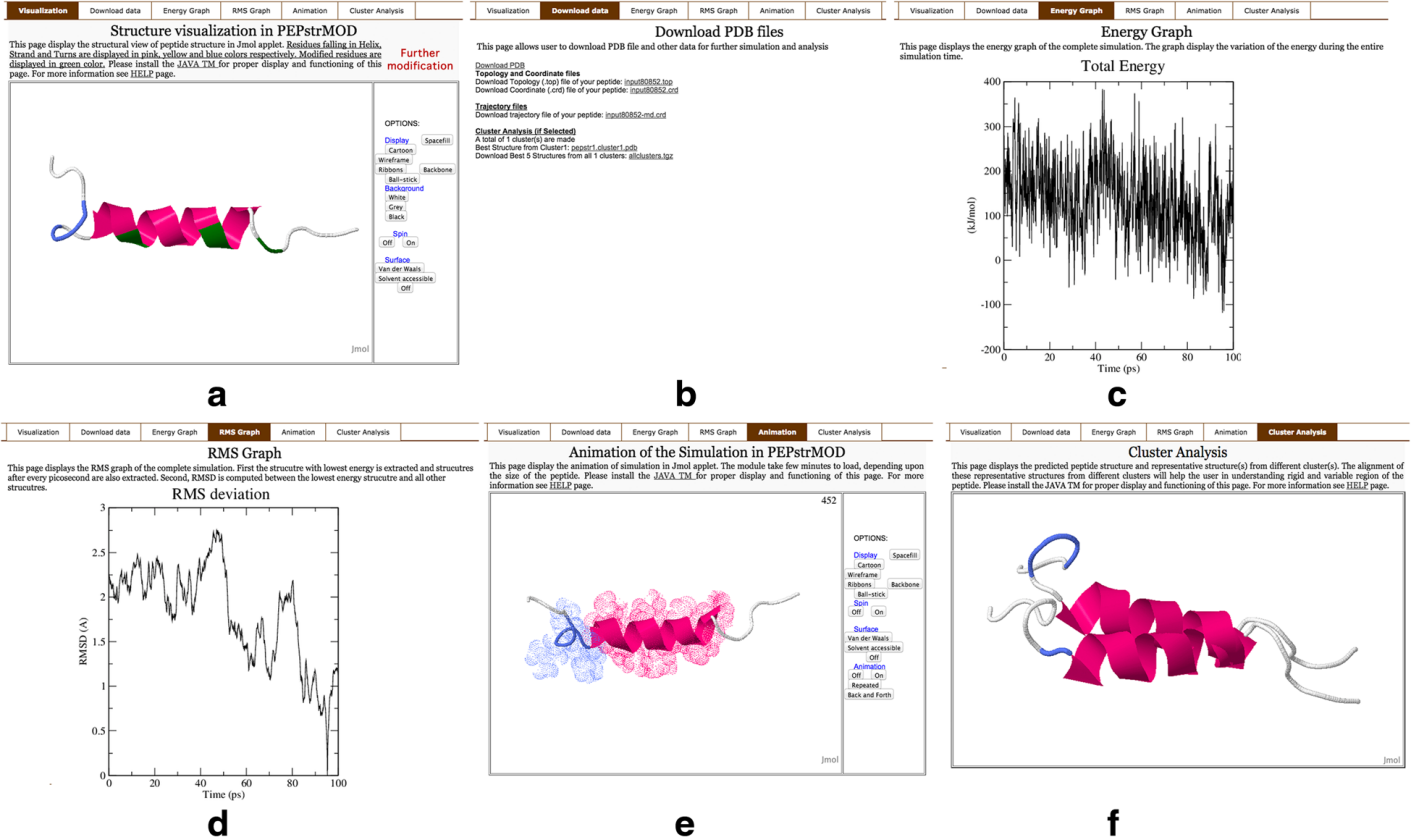
Graphical representation of the result page of PEPstrMOD with multiple tabs. **a** Visualization of the predicted structure using Jmol Viewer. **b** Links to download PDB file of predicted structure, topology, coordinate, trajectory files and representative structures from cluster analysis. **c** Energy graph of the simulation. **d** RMS graph of the simulation. **e** Visualization of the simulation in animated form. **f** Visualization of the alignment of predicted structure and representative structures obtained after cluster analysis

There are eight modules in PEPstrMOD, which can handle different modifications in a peptide. The name of these modules and their brief description is provided in Table 1. Each module has sub-modules, which can take either sequence or structure of the peptide as input. The ‘Structure Modification’ sub-module (implemented in all the modules) takes peptide structure as an input and gives an option to further modify the peptide structure. Other sub-modules, which take peptide sequence as input, are generally named either ‘Beginner’ or ‘Expert’ with the former being very easy to use. We have integrated different software and tools [43, 59, 60] to visualize and analyze the results obtained using PEPstrMOD (Fig. 3a, f). A detailed manual for using the online service of PEPstrMOD is available at (http://osddlinux.osdd.net/raghava/pepstrmod/pepstrmod_manual.pdf). PEPstrMOD web server can be accessed at http://osddlinux.osdd.net/raghava/pepstrmod/.

### Execution time

The total time required to predict the peptide structure using PEPstrMOD server depends on two factors. i) Environment (vacuum/hydrophilic/hydrophobic) in which the peptide needs to be simulated, which is selected by the user. In vacuum environment, at 100 ps MD simulation, the job is completed in ~5–8 min while in hydrophilic/hydrophobic environment the job can take up to ~45–60 min. ii) PEPstrMOD uses queue system where one query is processed at a time on ‘first-come, first-serve’ basis. To fasten the predictions, we have implemented three separate queue systems, one each for vacuum, hydrophilic and hydrophobic environments.

## Conclusions

Peptide-based therapeutics is currently being used for many diseases and disorders like diabetes, hypertension, heart attack, osteoporosis, cancer, hypothyroidism, acromegaly, infertility, bacterial infections, viral infections, etc. [2, 6, 39, 61, 62]. It becomes imperative to understand the structural and functional aspects of the peptides before undertaking systematic peptidomimetic approaches for designing therapeutic peptides with desirable properties [63–65]. In this direction, an attempt has been made to provide an online resource (PEPstrMOD) to predict the structure of a peptide from its sequence. Considering the importance of different types of modifications (cyclization, L- to D-conversion, non-natural residue, PTMs, capping of terminal residues), which are incorporated to provide therapeutic properties to the peptides, we integrated special force field libraries in PEPstrMOD to handle such peptides. We validated the PEPstrMOD application using three datasets and demonstrated that PEPstrMOD is able to approach near the native peptide structure. To the best of authors’ knowledge, PEPstrMOD is the only web server available currently to the scientific community, which can handle diverse modifications in peptides. We anticipate that PEPstrMOD will live up to the expectations of the researchers working in the field of peptide therapeutics/peptidomimetics.

### Limitations

The length range of the peptide, which can be modeled using PEPstrMOD, is 7 to 25 amino acids. PEPstrMOD can handle only those non-natural/modified amino acids, which are present in the force field libraries (FFNCAA/FFPTM/SwissSideChain) integrated in the PEPstrMOD. In future, with the availability of more force field libraries, it will be possible to include other non-natural modifications in PEPstrMOD.

## Reviewer’s comments

### Response to Prof. Michael Gromiha

In this work, the authors developed a method for predicting the structures of peptides containing natural and non-natural/modified residues. This program included six different types of peptides and desired modifications at any sites. The predicted structures showed a good agreement with experimental data. Further, a web server has been developed for peptide design. The server is well designed and sufficient details have been provided on the web with various utilities. The work is interesting and useful for designing peptides for desired therapeutic property. The following suggestions may be incorporated for improvements.

Comment 1: It will be better to avoid using abbreviations in the abstract.

Authors’ response 1: *In the revised manuscript, we have removed the abbreviations from the abstract.*

Comment 2: The source for obtaining experimental data for validating the method may be included in the abstract.

Authors’ response 2: *As suggested by the reviewer, we have provided more information on experimental data in the abstract.*

Comment 3: Page 6, psi value, −40 is split into two lines.

Authors’ response 3: *Correction has been made in the revised version of the manuscript.*

Comment 4: The time required to model the structure based on different types and number of modifications could be mentioned.

Authors’ response 4: *We have provided the approximate time required to build different types of models.*

Comment 5: Table 3, it will be helpful to write the explanations for the two methods in the footnote.

Authors’ response 5: *Suggestions of the reviewer have been incorporated in the revised version.*

Comment 6: In Table 2, it will be informative to give the meaning of the symbol B and the blank space.

Authors’ response 6: *Table**2**has been modified as suggested by the reviewer.*

Comment 7: The advantages of the present method over other related existing methods could be discussed.

Authors’ response 7: *We have discussed the advantages of the present method over existing methods in the revised manuscript.*

Comments from second revision: None

### Response to Dr. Bojan Zagrovic

The manuscript by Singh et al. presents a server for structure prediction of peptides with natural as well as non-natural/modified residues. Given the importance of natural and modified peptides in different areas of biomedicine and drug design, an effort in the direction of their 3D structure prediction just from sequence is definitely timely and highly needed. The server presented by the authors is well-designed and user friendly, but, as more elaborated below, I have reservations against it being a reliable structure prediction tool.

Authors’ response: *We are thankful to the reviewer for appreciating our work and his constructive comments. We have revised the manuscript and took following steps to address all the issues raised by the reviewer*.

(i) *We have made a large dataset of 501 peptides with different modifications and tested the performance of PEPstrMOD on this dataset rather than testing on 8 peptides.*
(ii) *Out of 501 peptides, we extracted 16 peptides which had regular secondary structure content ≥60 % and tested the performance of PEPstrMOD on these 16 peptides.*
(iii) *In order to demonstrate advantages of PEPstrMOD in building initial models, we also built structure of peptides using extended conformation. We compared the performance of models built using PEPstrMOD and extended conformations.*
(iv) *The performance of PEPstrMOD depends on predicted secondary structure so we also built models where we used actual secondary structure (generated using DSSP) instead of predicted secondary structure, to check the maximum performance, which can be achieved.*
(v) *We have performed ensemble-level comparison of the predicted structures with NMR rigid core regions. Rigid core regions are defined as the residues, which exhibit <1.5 Å CA-RMS fluctuations when all the models of the NMR structure are aligned (PMID 19569182).*
(vi) *We extended the MD simulations from 100 ps to 1 ns and evaluated the effect of MD simulations on accuracy of peptide structures.*
(vii) *We performed MD simulations in both vacuum and hydrophilic environment and compared the performance in both the environments.*

Major comments

Comment 1: The key part of the manuscript is the validation of the predictive capabilities of PEPstrMOD via a comparison between predicted and experimentally determined structures of 8 peptides containing non-natural residues and 4 pairs of peptides where both native and modified structures are known. This analysis, however, provides very little evidence that the algorithm is actually capable of quality predictions. For example, out of 8 peptides in Table 4, only 2 are predicted with a backbone RMSD < 5 Å, too few to give any strong credence to the claim that the algorithm can be used for structure prediction with any sort of reliability.

Authors’ response 1: *We agree with the reviewer that a small dataset of 8 peptides is not sufficient for demonstrating reliability of our method. Therefore, we replaced our small dataset with a large dataset having 501 peptides with different modifications (D-amino acids, non-natural residues and post-translational modifications). We evaluated the performance of PEPstrMOD on this dataset and achieved an average C-alpha RMSD (CA-RMSD) of 4.12 Å and Backbone-RMSD (B-RMSD) of 3.85 Å on this dataset. Out of 501 peptides, PEPstrMOD was able to achieve <5 Å B-RMSD in 387 peptides (~77 %). In the rigid core regions, PEPstrMOD achieved <5 Å B-RMSD in 399 peptides (~80 %) (Additional file**2**: Table S8). In the revised manuscript, we used the above dataset of 501 peptides for demonstrating the performance of our models*.

Comment 2: The latter point is easily understood if one consider the nature of the algorithm, which simply combines secondary structure/turn predictions based on primary sequence using PSIPRED and BetaTurns softwares with very short molecular dynamics (MD) runs (ca. 100 ps time-scale). On the one hand, the difficult part of structure prediction is done by PSIPRED and BetaTurns, but these algorithms are themselves fraught with uncertainties of multiple sorts. Putting these utilities together with an MD step into one framework is the main contribution of PEPstrMOD, but one should emphasize that on the 100 ps time-scale, as used by PEPstrMOD, MD is nothing more than a tool for local relaxation of structures, which in many cases (see next comment) actually lowers the accuracy of the prediction.

Authors’ response 2: *As pointed out by the reviewer, the initial structure of a peptide is built using the restraints predicted by PSIPRED and BetaTurns. These prediction methods have their own limitations. In order to demonstrate advantages and limitations of these secondary structure prediction methods; we also built models using actual secondary structure (DSSP generated) and extended conformations (no secondary structure information). We evaluated the performance of these models on a subset of 16 peptides extracted from 501 peptides that contain secondary structure content of 60 % or more. We evaluated the performance of these models on the initial structure, after energy minimization and after simulations at 100 ps and 1 ns. As shown in Additional file**2**: Table S9, we achieved CA-RMSD 10.94 Å, 4.83 Å and 2.78 Å for ab initio (extended conformation), PEPstrMOD (predicted secondary structure) and DSSP (actual secondary structure) based models respectively. These results clearly demonstrate advantages of PEPstrMOD over ab initio models, which is mainly due to the predicted secondary structure. The better performance of DSSP based methods demonstrates the limitations of the methods (PSIPRED & BetaTurns) used for predicting secondary structure*.

*Next, we computed the performance of models after energy-minimization and achieved CA-RMSD 10.77 Å, 4.78 Å and 2.90 Å for ab initio, PEPstrMOD and DSSP based models respectively. As shown in Additional file **2**: Table S9−S10, the performance of ab initio and PEPstrMOD improved slightly but it decreases slightly in case of DSSP-based model. Finally, we computed the performance of our models after performing MD simulations at 100 ps. It was observed that performance of DSSP-based models decreased after MD simulations. In case of PEPstrMOD, the performance of models improved after 100 ps simulation (CA-RMSD from 4.78–4.31 Å). Interestingly, the performance of ab initio models improved drastically from CA-RMSD 10.77–5.48 Å after 100 ps (Additional file **2**: Table S11). We also tested longer MD runs (up to 1 ns) and compared the performance of PEPstrMOD at both 100 ps and 1ns time steps. We didn’t observe any significant improvement in the results by extending the MD run from 100 ps to 1 ns (Additional file **2**: Table S12). This may be due to the fact that the initial structure predicted by PEPstrMOD is a good starting structure and a short MD simulation is sufficient to improve the structure.*

Comment 3: While the average RMSD over the whole test set presented in Table 4 does decrease after the molecular dynamics step, it turns out that the number of solid predictions (RMSD < 5 Å) actually drops from 4 to 2 (Table 4). The drop in the average RMSD is only due to an RMSD reduction for truly bad predictions (say, going from 14.56 Å to 8.20 Å for B–RMSD in the case of the 2CEF structure), but the key point is that 8 Å prediction is still as bad a prediction for a 19–residue peptide as is the 14 Å prediction. In contrast to the authors’ claim on p. 12, this in my opinion cannot be used to say anything about the quality of MD force fields in the task at hand.

Authors’ response 3: *We agree with the reviewer that PEPstrMOD completely fails to predict the structure of some peptides (RMSD >5 Å) but the performance was evaluated on a small dataset (8 peptides). In the revised manuscript we have validated PEPstrMOD method on a large dataset of 501 peptides with different modifications and achieved average CA-RMSD and B-RMSD of 4.12 Å and 3.85 Å respectively. Out of 501 peptides, PEPstrMOD was able to achieve <5 Å B-RMSD in 387 peptides (~77 %). In the rigid core regions, PEPstrMOD achieved <5 Å B-RMSD in 399 peptides (~80 %) (Additional file**2**: Table S8). In the revised manuscript, we have shown the performance of PEPstrMOD on a large dataset of 501 peptides.*

Comment 4: An important point when it comes to comparisons with experiment concerns the fact that most of the peptides are quite flexible, with the experimental NMR bundles in many cases exhibiting significant structural diversity. Therefore, even the whole concept of predicting the structure is undermined by the fact that many of these peptides do not have a fixed structure. Moreover, the authors in a way work against themselves when trying to compare their predictions against experiment, but only take into the account the first NMR model. An ensemble-level prediction and comparisons against experiment would be much more appropriate in this case.

Authors’ response 4: *As suggested by the reviewer, we have also performed ensemble-level comparisons of the predicted structures with NMR rigid core regions. Rigid core regions are defined as the residues, which exhibit <1.5 Å CA-RMS fluctuations when all the models of the NMR structure are aligned (PMID 19569182).*

Comment 5: The comparison of the predicted structures of cyclized peptides with their experimentally determined counterparts shows that PEPstrMOD performs quite well and with a similar level of accuracy as the competitor PEP-FOLD. However, one might argue that the moment one connects the correct cysteine residues in peptides via S-S bridges, the near-correct topology is immediately attained, especially for very short peptides. The authors should discuss this fact in more detail, and also present a more thorough analysis of the cases where their predictions failed (e.g. in about 1/3 of cases their backbone RMSDs from the correct structure > 5 Å).

Authors’ response 5: *We agree with the reviewer that cyclic peptides have limited conformations as S-S bridge may provide near-correct topology in the case of small peptides. In order to demonstrate advantage of PEPstrMOD in predicting structure of cyclic peptides, we also predicted the structure of cyclic peptides using ab initio model where structure is built from extended conformation using disulfide bond constraints and subjected to MD simulations. As shown in Table**3**, the performance of PEPstrMOD model is better than ab initio models. PEPstrMOD achieved an average CA-RMSD of 4.06 Å where as ab initio model achieved an average CA-RMSD of 5.04 Å after MD simulation of 100 ps. In the revised manuscript, we have discussed this fact in detail and have also provided a thorough analysis of the cases where PEPstrMOD performance was poor.*

Comment 6: The analysis that has lead to the data given in Table 5 is not clearly explained. What do the authors mean, when they say that “structure of original peptide was used” (p. 12)? The authors should also provide a comparison between the predicted and the actual structures of the modified peptides without using the non-modified experimental structure of the peptide as template.

Authors’ response 6: *The dataset used in Table**5**is very small (4 pairs of peptides) and we agree with the reviewer that validating any method on a very small dataset doesn’t ensure the reliability of the method. Therefore, in the revised manuscript we have now removed the results on this dataset i.e. Table**5**. We have validated PEPstrMOD on a new and large dataset of 501 peptides.*

Comment 7: Overall, the webpage of the server is nicely designed and implemented and one must appreciate the amount of work that has gone into it. However, for the reasons outlined above, I see its utility much less as a peptide structure prediction engine in the strict sense, but more as a useful tool for generating plausible starting structures for MD simulations of modified peptides. I doubt that, due to the inaccuracies involved, the structures obtained by the server will have any strong immediate practical value when it comes to drug design, docking or structure modeling, except perhaps in the case of cyclized molecules or as a starting point for further studies.

Authors’ response 7: *In the revised manuscript we have validated the PEPstrMOD method on a large dataset of 501 peptides. Considering the challenging task of peptide tertiary structure prediction directly from sequence, PEPstrMOD achieved reasonable performance. We hope that the revised manuscript will address the concerns raised by the reviewer.*

Minor comments

Comment 8: The discussion of different algorithms for peptide structure prediction given in the introduction should include a more critical comparison of the advantages and disadvantages of different algorithms and not just a sheer listing of their names and basic properties.

Authors’ response 8: *We have modified the introduction and added the content as per the suggestion of the reviewer.*

Comment 9: The quality of the written English in the manuscript as whole is good, but the text could still benefit from a round of editing. In particular, there is a number of places where definite or indefinite articles are missing. Also, values of different variables and the associated units should be separated. Finally, Table 2 should be edited – currently some cells are filled with non-standard characters whose meaning is difficult to decipher.

Authors’ response 9: *We have made appropriate changes in the revised manuscript wherever required. We are thankful to the reviewer for his suggestions that helped us to improve the manuscript. In the revised manuscript, we have corrected Table**2*.

Comment 10: While the reference to the PTM parameters for GROMOS 45a3 and 54a7 is correct (ref. 37), the reference to the Vienna PTM web server should be included as: Margreitter et al. Nucleic Acids Research, 41, Web Server Issue, W422–W426.

Authors’ response 10: *In the revised manuscript, we have corrected the reference to the Vienna PTM web server.*

Comment 11: Description of the comparison for cycled peptides is unclear - did or did not the authors include disulfide bond constraints with PEP-FOLD?

Authors’ response 11: *Disulfide bond constraints were included with PEP-FOLD and in the manuscript it is represented by PEP-FOLD*^*c*^*where superscript ‘c’ stands for ‘constraints’*.

### Comments from second revision

The manuscript by Singh et al. presents a server for structure prediction of peptides with natural as well as non-natural/modified residues. Given the importance of natural and modified peptides in different areas of biomedicine and drug design, an effort in the direction of their 3D structure prediction just from sequence is definitely timely and highly needed. The server presented by the authors is well designed and user friendly, and has been validated and tested on a large dataset of 501 peptides.

While the performance of the algorithm as demonstrated in the article is good, there are several points which should be borne in mind when using it, and which could in some (but, not all) applications lead to difficulties. Namely, the algorithm combines secondary structure/turn predictions based on primary sequence using PSIPRED and BetaTurns software packages with very short molecular dynamics (MD) runs (100 ps time-scale). On the one hand, the difficult part of structure prediction is done by PSIPRED and BetaTurns, but these algorithms are themselves fraught with uncertainties of multiple sorts. Putting these utilities together with an MD step into one framework is the main contribution of PEPstrMOD, but one should emphasize that on the 100 ps time-scale, as used by PEPstrMOD, MD is nothing more than a tool for local relaxation of structures, which in some cases could actually lower the accuracy of the prediction. Finally, an important point when it comes to comparisons with experiment concerns the fact that many peptides are quite flexible, with the experimental NMR bundles in many cases exhibiting significant structural diversity. Therefore, even the whole concept of predicting the structure may be undermined by the fact that many of these peptides do not have a fixed structure. While the authors clearly show that the structures of the more stable and rigid parts of peptides can be accurately predicted, practical users should keep in mind that the more flexible parts might simply be floppy and could only be accurately described by using extensive MD simulations or similar methods. In this sense, I see the utility of the algorithm both as a peptide structure prediction engine, but also as a useful tool for generating plausible starting structures for MD simulations of modified peptides.

Authors’ response: *We are thankful to the reviewer for appreciating our work. The comments and suggestions of the reviewers helped us to improve our work.*

### Response to Dr. Zoltan Gaspari

The authors describe a web service for structure prediction of peptides containing modified/unnatural amino acids. They developed a pipeline using AMBER or GROMACS, depending on the available force field parameters for the given amino acids in the peptide. I feel that the concept is useful but I am not fully satisfied with the presentation of the results. In particular, I would like to ask the authors to address the following points:

Authors’ response: *We are thankful to the reviewer for appreciating our work and his constructive comments. We have revised the manuscript and took following steps to address all the issues raised by the reviewer.*

(i) *We have made a large dataset of 501 peptides with different modifications and tested the performance of PEPstrMOD on this dataset rather than testing on 8 peptides.*
(ii) *Out of 501 peptides, we extracted 16 peptides which had regular secondary structure content ≥60 % and tested the performance of PEPstrMOD on these 16 peptides.*
(iii) *In order to demonstrate advantages of PEPstrMOD in building initial models, we also built structure of peptides using extended conformation. We compared the performance of models built using PEPstrMOD and extended conformations.*
(iv) *The performance of PEPstrMOD depends on predicted secondary structure so we also built models where we used actual secondary structure (generated using DSSP) instead of predicted secondary structure, to check the maximum performance, which can be achieved.*
(v) *We have performed ensemble-level comparison of the predicted structures with NMR rigid core regions. Rigid core regions are defined as the residues, which exhibit <1.5 Å CA-RMS fluctuations when all the models of the NMR structure are aligned (PMID 19569182).*
(vi) *We extended the MD simulations from 100 ps to 1 ns and evaluated the effect of MD simulations on accuracy of peptide structures.*
(vii) *We performed MD simulations in both vacuum and hydrophilic environment and compared the performance in both the environments.*

Comment 1: I would like to see more details about the conformation assigned to the ‘X’ amino acids. For example, for residues in the D configuration, are the L-counterparts taken into account or are they also treated as ‘X’? It would be worth to analyze how this influences the prediction of secondary structures and to what extent the differences between predicted and actual structures - where it can be assessed - come from erroneous assignment of the starting conformation of the ‘X’ residue.

Authors’ response 1: *Only non-natural amino acids are converted to ‘X’ amino acid prior to secondary structure prediction step using PSIPRED. Therefore, residues in D configuration are not converted to ‘X’. Rather handling of D-amino acids is done using AMBER command ‘flip’ which flips the stereochemistry of the selected residue. Similarly, residues with post-translational modifications (like 5hydroxy-lysine) are not converted into ‘X’ and the original residue is retained as such, prior to PSIPRED step. This is also represented in Fig.**1**where residue lysine is retained as such, prior to PSIPRED step, while non-natural residue diethylalanine is converted to ‘X’.*

Comment 2: In connection, it would be useful to include/refer to a critical evaluation of the incorporated secondary structure prediction methods for some unmodified peptides. Also, I am not fully convinced that the backbone torsion angles 180/180 can be referred to as ‘ideal’ for ‘coil’ residues as ‘coil’ refers to everything that is not alpha-helix of beta-sheet in this context.

Authors’ response 2: *The effect of incorporation of predicted secondary structure restraints in the prediction method for unmodified peptides has already been shown in the PEPstr algorithm [PMID 17897087]. Briefly, after applying the secondary structure restraints to the extended conformation, followed by energy minimization, the RMSD decreased from 7.1 Å to 4.4 Å.*

*As pointed out by the reviewer, to demonstrate the advantages and the limitations of these secondary structure prediction methods; we also built models using actual secondary structure (DSSP generated) and extended conformations (no secondary structure information). We evaluated the performance of these models on a subset of 16 peptides extracted from 501 peptides that contain secondary structure content of 60 % or more. We evaluated the performance of these models on initial structure, after energy minimization and after simulations at 100 ps and 1 ns. As shown in Additional file **2**: Table S9, we achieved CA-RMSD 10.94 Å, 4.83 Å and 2.78 Å for ab initio (extended conformation), PEPstrMOD (predicted secondary structure) and DSSP (actual secondary structure) based models respectively. These results clearly demonstrate the advantages of PEPstrMOD over ab initio models, which is mainly due to the predicted secondary structure. The better performance of DSSP based methods demonstrates the limitations of methods (PSIPRED & BetaTurns) used for predicting secondary structure.*

*Next, we computed the performance of models after energy-minimization and achieved CA-RMSD 10.77 Å, 4.78 Å and 2.90 Å for ab initio, PEPstrMOD and DSSP based models respectively. As shown in Additional file **2**: Table S9−S10, the performance of ab initio and PEPstrMOD improved slightly but it decreases slightly in the case of DSSP-based model. Finally, we computed the performance of our models after performing MD simulations at 100 ps. It was observed that the performance of DSSP-based models decreased after MD simulations. In case of PEPstrMOD, the performance of models improved after 100 ps simulation (CA-RMSD from 4.78–4.31 Å). Interestingly, the performance of ab initio models improved drastically from CA-RMSD 10.77–5.48 Å after 100 ps (Additional file **2**: Table S11). We also tested longer MD runs (up to 1 ns) and compared the performance of PEPstrMOD at both 100 ps and 1 ns time steps. We didn’t observe any significant improvement in the results by extending the MD run from 100 ps to 1 ns (Additional file **2**: Table S12). This may be due to the fact that the initial structure predicted by PEPstrMOD is a good starting structure and a short MD simulation is sufficient to improve the structure.*

*We agree with the reviewer that the backbone torsion angles 180/180 cannot be referred to as ideal for coil residues, instead they are used to make an extended (linear) conformation. In the revised manuscript, we have modified the statement and have added the details of the above experiment along with its discussion.*

Comment 3: In the evaluation of correspondence of predicted vs, experimental structures I would like to see the final predicted torsion angles as they might be more informative than RMSD values. in particular, even if an RMSD is relatively high, some important aspects of the structure, the presence of an important local structural motif might be predicted accurately. For structures where NMR restraints are available it could be also useful to analyze the correspondence of the predicted structures to these.

Authors’ response 3: *As suggested by the reviewer, we have now performed ensemble-level comparison of the predicted structures with NMR rigid core regions. Rigid core regions are defined as the residues, which exhibit <1.5 Å CA-RMS fluctuations when all the models of the NMR structure are aligned (PMID 19569182). Therefore, comparison of the structures only in the rigid core regions provides more detailed analysis of the prediction methods.*

Comment 4: The limitations of the method should be discussed, i.e. what is the maximum length of a peptide that can be predicted within a reasonable time and how many nonstandard residues are allowed. E.g. are two consecutive modified residues allowed/expected to be modeled accurately?

Authors’ response 4: *In the revised manuscript we have discussed the limitations of the methods with respect to the maximum length of the peptide that can be predicted. An approximate time required to model the structure with different modifications is also discussed.*

*Two consecutive modified residues can be modeled using PEPstrMOD. Moreover, the newly created dataset of 501 peptides on which PEPstrMOD is validated, contains such entries with two and even three consecutive modified residues. However, such entries with consecutive residues are less in number (26 out of 501). PEPstrMOD also provides the provision to model multiple non-consecutive modified residues, which are also present in the new dataset of 501 peptides.*

Comment 5: Have the authors tested longer MD runs/simulated annealing protocols? Please comment on this whether any of these could be expected to improve the results.

Authors’ response 5: *As per the suggestion of the reviewer, we tested longer MD runs (up to 1 ns) and compared the performance of PEPstrMOD. We observed no significant improvement in the results by extending MD simulation up to 1 ns. Briefly, on ‘Cyclicpep’ dataset (34 cyclic peptides), PEPstrMOD achieved an average CA-RMSD of 4.06 Å and 4.10 Å at 100 ps and 1 ns time steps respectively (Additional file**2**: Table S5). However, repeating the same experiment while performing MD simulation in hydrophilic environment, a slight improvement was observed in the performance of PEPstrMOD from 3.97 Å to 3.82 Å at 100 ps and 1 ns time steps respectively (Additional file**2**: Table S6).*

*On 16 peptides (with regular secondary structure content ≥60 %), PEPstrMOD achieved an average CA-RMSD of 4.31 Å and 4.48 Å at 100 ps and 1 ns time steps respectively. Performing same experiment with MD simulation in hydrophilic environment, PEPstrMOD achieved average CA-RMSD of 4.35 Å and 4.36 Å respectively (Additional file **2**: Table S11−S12, S13-S14).*

### Comments from second revision

The authors have put a lot of work into improving the manuscript compared to the initial version and I appreciate their efforts. The authors have extended their comparison methodology even though I did not get a direct answer for my point about torsion angles and NMR restraints. I think that the manuscript is now suitable for publication.

Authors’ response: *We are thankful to the reviewer for appreciating our work. The comments and suggestions of the reviewers helped us to improve our work*.

## Additional files

Additional file 1:
**List of modified residues integrated in PEPstrMOD; AMBER and GROMACS parameters for performing Molecular Dynamics.** **Table S1.** Types of non-natural amino acids present in FFNCAA and SwissSideChain Force field libraries. **Table S2.** Types of PTMs available in FFPTM force field library. **Table S3.** AMBER parameters for performing energy minimization and molecular dynamics using AMBER11. **Table S4.** GROMACS parameters for performing energy minimization and molecular dynamics using GROMACS-4.6.5. (PDF 50 kb) Additional file 2:
**Performance of PEPstrMOD on CyclicPep, ModPep and ModPep16 datasets.** **Table S5.** Performance comparison of PEPstrMODpc with PEP-FOLDc and ab initio model on CyclicPep dataset in vacuum environment. **Table S6.** Performance comparison of PEPstrMODpc and ab initio model on CyclicPep dataset in hydrophilic environment. **Table S7.** Performance of PEPstrMOD, PEP-FOLD and PepLook on 28 cyclic peptides. **Table S8.** Performance of PEPstrMOD on ModPep dataset. **Table S9.** Comparison of the performance of initial structures of PEPstrMOD, ab initio and DSSP models on ModPep16 dataset. **Table S10.** Comparison of the performance of minimized structures of PEPstrMOD, ab initio and DSSP models on ModPep16 dataset in vacuum environment. **Table S11.** Comparison of the performance of structures of PEPstrMOD, ab initio and DSSP models on ModPep16 dataset in vacuum environment after 100 ps MD. **Table S12.** Comparison of the performance of structures of PEPstrMOD, ab initio and DSSP models on ModPep16 dataset in vacuum environment after 1 ns MD. **Table S13.** Comparison of the performance of minimized structures of PEPstrMOD, ab initio and DSSP models on ModPep16 dataset in hydrophilic environment. **Table S14.** Comparison of the performance of structures of PEPstrMOD, ab initio and DSSP models on ModPep16 dataset in hydrophilic environment after 100 ps MD. **Table S15.** Comparison of the performance of structures of PEPstrMOD, ab initio and DSSP models on ModPep16 dataset in hydrophilic environment after 1 ns MD. (XLSX 176 kb)

## Abbreviations

B-RMSD: Backbone root mean square deviation
CA-RMSD: C-alpha root mean square deviation
FFNCAA: Forcefield_NCAA
FFPTM: Forcefield_PTM
MD: Molecular dynamics.
ns: Nanosecond
ps: Picosecond
RC Region: Rigid core region
RMSD: Root mean square deviation
