# Supplementary material for: PEPstrMOD: structure prediction of peptides containing natural, non-natural and modified residues
Source: Biol Direct. 2015 Dec 21;10:73. doi: 10.1186/s13062-015-0103-4 (PMC4687368; doi:10.1186/s13062-015-0103-4)
Supplement: Additional file 1: — List of modified residues integrated in PEPstrMOD; AMBER and GROMACS parameters for performing Molecular Dynamics. Table S1. Types of non-natural amino acids present in FFNCAA and SwissSideChain Force field libraries. Table S2. Types of PTMs available in FFPTM force field library. Table S3. AMBER parameters for performing energy minimization and molecular dynamics using AMBER11. Table S4. GROMACS parameters for performing energy minimization and molecular dynamics using GROMACS-4.6.5. (PDF 50 kb) [file 13062_2015_103_MOESM1_ESM.pdf]

PEPstrMOD: Structure Prediction of Peptides Containing Natural, Non-natural and Modified Residues.

Sandeep Singh[1], Harinder Singh[1], Abhishek Tuknait[1], Kumardeep Chaudhary[1], Balvinder Singh[1], S Kumaran[2] and Gajendra P.S. Raghava[1]*

[1]Bioinformatics Centre, CSIR-Institute of Microbial Technology, Chandigarh-160036, India

[2]CSIR-Institute of Microbial Technology, Chandigarh-160036, India

ADDITIONAL FILE 1: List of modified residues integrated in PEPstrMOD; AMBER and GROMACS parameters for performing Molecular Dynamics.

TableS1: Types of non-natural amino acids present in FFNCAA and SwissSideChain Force field libraries

Parent Amino acid	Non-natural modifications present in FFNCAA	Non-natural modifications present in SwissSideChain
ALA	3-cyclopentylalanine, diethylalanine, Cyclohexylalanine, 1-naphthylalanine, 2-naphthylalanine, 5-hydroxy-1-naphthylalanine, 6-hydroxy-2-naphthylalanine, 3-(9-anthryl)-alanine, 3-(2-pyridyl)-alanine, 3-(3-pyridyl)-alanine, 3-(4-pyridyl)-alanine, 3-(2-quinolyl)-alanine, 3-(3-quinolyl)-alanine, 3-(4-quinolyl)-alanine, 3-(5-quinolyl)-alanine, 3-(6-quinolyl)-alanine, 3-(8-hydroxyquinolin-3-yl)-alanine, N-methylalanine, 1-pyrenylalanine, (R)-alpha-ethylalanine, (S)-alpha-ethylalanine, R(+)-alpha-Allylalanine, (R)-2,(2'-propenyl)alanine, (R)-2,(4'-pentenyl)alanine, (R)-2,(7'-octenyl)alanine, beta-alanine	3-(4H-thieno[3,2-b]pyrrol-6-yl)-L-alanine, 3-cyclohexyl-alanine, m-amidinophenyl-3-alanine, azido-alanine, 3-(3-benzothienyl)-alanine, 3-chloro-l-alanine, 3-Cyclopentyl-alanine, 3,3-dihydroxy-alanine, Trifluoro-alanine, (2-furyl)-alanine, Styrylalanine, 3-(2-Tetrazolyl)-alanine, 3-(3-thienyl)-alanine, 3-(2-thienyl)-alanine, 3-(1,2,4-Triazol-1-yl)-alanine, (4-thiazolyl)-alanine
CYS	(R)-L-alpha-methylcysteine, cysteine acetamide, N-methylcysteine, carboxymethylated cysteine, benzyl cysteine, (S)-(2-hydroxyethyl)-cysteine, (S)-acetonylcysteine, beta-cysteine	s-(difluoromethyl)-homocysteine, Selenocysteine, homocysteine
ASP	N-methylaspartic acid, 3-methyl-aspartic acid, beta-aspartic-acid	beta-hydroxyaspartic acid, 3,3-dimethyl aspartic acid

GLU	5-o-methyl-glutamic acid, (2S,4R)-4-methylglutamate, N-methyl-glutamicacid, (3R)-3-methyl-glutamic acid, (3S)-3-methyl-glutamic acid	4-hydroxy-glutamic-acid, 4-Fluoro-glutamic acid
PHE	alpha-methyl-l-phenylalanine, 2-ethyl,4-o-methyl-biphenylalanine, 3-methyl-biphenylalanine, 3-o-methyl-biphenylalanine, 2-ethyl-biphenylalanine, 2-methyl-4-o-methyl-biphenylalanine, 2-methyl-biphenylalanine, biphenylalanine, 2-methylphenylalanine, 3-methylphenylalanine, 4-methylphenylalanine, 4-tert-butyl-phenylalanine, 4-amino-phenylalanine, 4-methoxy-phenylalanine	4-carbamimidoyl-l-phenylalanine, 3-cyano-phenylalanine, 4-Bromo-phenylalanine, 4-cyano-phenylalanine, 4-hydroxymethyl-phenylalanine, 4,4-biphenylalanine, 2-cyano-phenylalanine, 2,4-dichloro-phenylalanine, 3,4-dichloro-phenylalanine, 3,4-Dihydroxy-phenylalanine, 3,3-diphenylalanine, 3-ethyl-phenylalanine, 3,4-Difluoro-phenylalanine, 3,4-Dimethyl-phenylalanine, 4-benzoyl-phenylalanine, pentafluoro-phenylalanine, 4-Nitro-phenylalanine, 2-(Trifluoromethyl)-phenylalanine, 3-(Trifluoromethyl)-phenylalanine, 4-(Trifluoromethyl)-phenylalanine, 3,5-Difluoro-phenylalanine
GLY	(2-indanyl)-glycine, vinylglycine, phenylglycine, 4-hydroxyphenylglycine, N-methylglycine, 2-allyl-glycine, beta-glycine	Cyclohexylglycine, 3-chloro-4-hydroxy-phenylglycine, 2-Chloro-phenylglycine, 3-Chloro-phenylglycine, 4-Chloro-phenylglycine, 2-Fluoro-phenylglycine, 3-Fluoro-phenylglycine, 4-Fluoro-phenylglycine, 2-(Trifluoromethyl)-phenylglycine, 3-(Trifluoromethyl)-phenylglycine, 4-(Trifluoromethyl)-phenylglycine, 2-thienylglycine, 3-thienylglycine
HIS	N-methyl-histidine	
ILE	N-methylisoleucine, allo-isoleucine, 3-methyl-alloisoleucine, beta-isoleucine	5-bromo-l-isoleucine, 4-Hydroxy-L-isoleucine, 3-methyl-l-alloisoleucine, 4,5-dihydroxy-isoleucine
LYS	N-methyl-lysine, beta-lysine	beta-lysine, 6-carboxylysine, thialysine

LEU	N-methyl-leucine, homoleucine, 6-hydroxy-norleucine, norleucine, (R)-alpha-methylleucine, (tert-butyl)leucine, beta-hydroxyleucine	(4r)-5-oxo-l-leucine, Tertleucine, (4s)-5-fluoro-l-leucine, 5-oxo-l-norleucine, 5,5,5-Trifluoro-leucine
MET	hydroxyl-methionine, ethionine, N-methyl-methionine, beta-methionine	nitrilo-l-methionine, 3,3-dimethyl-methionine sulfoxide, s-oxymethionine
ASN	N4-methyl-asparagine, N-methyl asparagine	beta-hydroxyasparagine
PRO	NA	NA
GLN	N-methylglutamine, N5-methyl-glutamine, glutamine hydroxamate, 3-methyl glutamine, beta-glutamine	NA
ARG	N-methyl-arginine	5-methyl-arginine, c-gamma-hydroxy arginine, homoarginine
SER	Homoserine, N-methyl-serine, beta-serine	Phenylserine, o-acetylserine, phosphoserine, phosphoserine(charge:-2),
THR	o-methyl-threonine, beta-threonine	Allo-threonine, 4-chloro-threonine, 4-hydroxy-l-threonine, phosphothreonine, phosphothreonine(charge:-2)
VAL	(R)-(+)-alpha-methylvaline, norvaline, N-methyl-valine, hydroxynorvaline, beta-valine	3-Fluoro-valine, 3-hydroxy-l-valine, 4-oxo-l-valine
TRP	5-methyltryptophan, 1-methyltryptophan, N-methyltryptophan, 2-hydroxy-tryptophan, 4-amino-tryptophan, 6-methyltryptophan, 5-methoxytryptophan, beta-hydroxy-tryptophane, 5-hydroxytryptophan, beta-tryptophan	4-fluoro-tryptophan, 4-hydroxy-tryptophan, 6-chloro-l-tryptophan, 6-bromo-tryptophan, 7-chloro-tryptophan, 6-fluoro-l-tryptophan, 5-Fluoro-tryptophan, 5-Methyl-tryptophan, 6-hydroxy-tryptophan, 6-amino-7-hydroxy-l-tryptophan
TYR	O-methyltyrosine, O-ethyltyrosine, O-allyltyrosine, N-methyltyrosine, o-tyrosine, 3-amino-tyrosine, 3-amino-6-tyrosine, (beta)-beta-hydroxy-tyrosine, beta-tyrosine	3-Chloro-tyrosine, 3,5-dibromotyrosine, 3-iodo-tyrosine, meta-nitro-tyrosine, (betar)-3-chloro-beta-hydroxy-l-tyrosine, phosphotyrosine, phosphotyrosine(charge:-2), 3-Amino-L-tyrosine, 3,5-diiodotyrosine, 3-fluorotyrosine
Others	Alpha-aminobutyric acid, 2-aminobutyric acid, adamanthane, 2-	2-amino-6-oxopimelic acid, l-2-amino-6-

	aminoheptanoic acid, (2s,4s)-2,5-diamino-4-hydroxy-5-oxopentanoic acid, 2-amino-propanedioic acid, 2-amino-6-oxopimelic acid, (2S)-amino(3,5-dihydroxyphenyl)-ethanoic acid, kynurenine, (R)-alpha-methylornithine, (S)-alpha-methylornithine, 2,3-diaminopropanoic acid, diaminobutyric acid, (2S)-2,8-diaminooctanoic acid, ethionine, 2-amino-5-hydroxypentanoic acid, 6-hydroxy-norleucine, Ornithine	methylene-pimelic acid, cis-amiclenomycin, (2r)-2-amino-4-oxobutanoic acid, canaline, Citrulline, 2-Amino-4-guanidinobutryric acid, 2-Amino-3-guanidinopropionic acid, Canavanine, (2s,3r)-2-amino-3-hydroxy-4-methylpentanoic acid, alpha-amino-2-indanacetic acid, Penicillamine, 5-hydroxy-1-naphthalene, 6-hydroxy-2-naphthalene, o-acetylserine, (2s)-2-amino-4,4-difluorobutanoic acid, Thio-citrulline, 2-Aminoadipic acid

TableS2: Types of PTMs available in FFPTM force field library.

Parent Amino Acid	PTMs present in FFPTM
ALA	NA
CYS	Cysteinepersulfide, Cysteine sulfenic acid, Phosphocysteine (neutral), S-farnesylcysteine, S-geranylgeranylcysteine, S-palmitoylcysteine
ASP	NA
GLU	1-Carboxyglutamic Acid (neutral)
PHE	Dihydroxyphenylalanine
GLY	NA
HIS	NA
ILE	NA
LYS	5-hydroxylysine, epsilon-N-methyllysine, N-epsilon-acetyllysine, N6N6-dimethyllysine, N6N6N6-trimethyllysine, Pyrrolysine, N6propanoyllysine, N6butanoyllysine
LEU	N-methyl-leucine, homoleucine, (R)-alpha-methylleucine, beta-hydroxyleucine
MET	hydroxyl-methionine, ethionine, N-methyl-methionine, beta-methionine

ASN	NA
PRO	3,4-hydroxyproline, 3-hydroxyproline, 4-hydroxyproline
GLN	NA
ARG	Dimethylarginine (Anti-symmetric), (omega)-methylarginine
SER	Phosphoserine (neutral), Phosphoserine (-2 charge)
THR	Phosphothreonine (neutral), Phosphothreonine (-2 charge)
VAL	NA
TRP	7-hydroxytryptophan, 5-hydroxytryptophan
TRY	Phosphotyrosine (neutral), Phosphotyrosine (-2 charge)
Others	Ornithine

Table S3. AMBER parameters for performing energy minimization and molecular dynamics using AMBER11.

**Vacuum Environment**		
Energy Minimization		imin=1, ntmin=1, maxcyc=2000, ncyc=1000, ntb=0, igb=0, cut=10.0
Molecular Dynamics (MD)	Heating	imin=0, irest=0, ntx=1, ntb=0, ntc=2, ntf=2, cut=10.0, igb=0, tempi=0, temp0=300, ntt=3, gamma_ln=2, nstlim=50000, dt=.001,ig=-1, ntpr=100, ntwx=100
	Production MD	imin=0, irest=0, ntx=5, ntb=0, ntc=2, ntf=2, cut=10.0, igb=0, tempi=300, temp0=300, ntt=3, gamma_ln=2, nstlim=100000, dt=.001,ig=-1, ntpr=100, ntwx=100
**Hydrophilic/Hydrophobic Environment**		
Energy Minimization		imin=1, maxcyc=2000, ntmin=1, ncyc=1000, ntb=1,cut=10.0
Molecular Dynamics	Heating (NVT) ensemble	imin=0,irest=0,ntx=1, ntb=1,ntc=2,ntf=2, cut=10.0, tempi=0,temp0=300,ntt=3,gamma_ln=2, nstlim=50000,dt=.001,ig=-1, ntpr=100,ntwx=100
	NPT ensemble	imin=0, irest=1, ntx=5,ntr=0, ntb=2, taup=2, pres0=1, ntp=1, ntc=2, ntf=2, cut=10.0, tempi=300,temp0=300,ntt=3, gamma_ln=2, nstlim=50000, dt=.001,ig=-1, ntpr=100, ntwx=100
	Production MD	imin=0, irest=1, ntx=5,ntr=0, ntb=2, taup=2, pres0=1, ntp=1, ntc=2, ntf=2, cut=10.0, tempi=300,temp0=300,ntt=3, gamma_ln=2, nstlim=100000, dt=.001,ig=-1, ntpr=100, ntwx=100

Table S4. GROMACS parameters for performing energy minimization and molecular dynamics using GROMACS-4.6.5.

**Vacuum Environment**		
Energy Minimization		integrator=cg, emtol=100.0, emstep=0.01, nsteps=2000, nstcgsteep=1000, pbc=no
Molecular Dynamics (MD)	Heating	integrator=md, nsteps=50000, dt=0.001, nstxout=500, nstvout=500, nstenergy=500, nstxtcout=500, nstlog=500, energygrps=Protein, continuation=no, constraint_algorithm=lincs, constraints=h-bonds, vdwtype=Cut-off, nstlist=0, ns_type=simple, pbc=no, rlist=0.0, rlistlong=0.0, coulombtype=Cut-off, rcoulomb-switch=0, rcoulomb=0.0, rvdw=0.0, rvdw-switch=0.0, tcoupl=V-rescale, tc-grps=Protein, tau_t=0.1, ref_t=300, pcoupl=no, gen_vel=yes, gen_temp=0, gen_seed=-1
	Production MD	integrator=md, nsteps=100000, dt=0.001, nstxout=500, nstvout=500, nstenergy=500, nstxtcout=500, nstlog=500, energygrps=Protein, continuation=no, constraint_algorithm=lincs, constraints=h-bonds, vdw-type=Cut-off, nstlist=0, ns_type=simple, pbc=no, rlist=0.0, rlistlong=0.0, coulombtype=cut-off, rcoulomb-switch=0, rcoulomb=0.0, rvdw=0.0, rvdw-switch=0.0, tcoupl=V-rescale, tc-grps=Protein, tau_t=0.1, ref_t=300, pcoupl=no, gen_vel=no
**Hydrophilic/Hydrophobic Environment**		
Energy Minimization		integrator=cg, emtol=100.0, emstep=0.01, nsteps=2000, nstcgsteep=1000 nstlist=1, ns_type=grid, coulombtype=PME
Molecular Dynamics	Heating (NVT) ensemble	integrator=md, nsteps=50000, dt=0.001, nstxout=100, nstvout=100, nstenergy=100, nstlog=100, continuation=no, constraint_algorithm=lincs, constraints=h-bonds, ns_type=grid, nstlist=10, coulombtype=PME, tcoupl=V-rescale, tc-grps=Protein Non-Protein, tau_t=0.1 0.1, ref_t=300 300, pcoupl=no, pbc=xyz, DispCorr=EnerPres, gen_vel=yes, gen_temp=0, gen_seed=-1
	NPT ensemble	integrator=md, nsteps=50000, dt=0.001, nstxout=100, nstvout=100, nstenergy=100, nstlog=100, continuation=yes, constraint_algorithm=lincs, constraints=h-bonds, ns_type=grid, nstlist=10, coulombtype=PME, tcoupl=V-rescale, tc-grps=Protein Non-Protein, tau_t=0.1 0.1, ref_t=300 300, pcoupl=Parrinello-Rahman, pcoupltype=isotropic, tau_p=2.0, ref_p=1.0, compressibility=4.5e-5, refcoord_scaling=com, pbc=xyz, DispCorr=EnerPres, gen_vel=no
	Production MD	integrator=md, nsteps=100000, dt=0.001, nstxout=500, nstvout=500, nstxtcout=500, nstenergy=500, nstlog=500, continuation=yes, constraint_algorithm=lincs, constraints =h-bonds, ns_type=grid, nstlist=10, coulombtype=PME, tcoupl=V-rescale, tc-grps=Protein

		Non-Protein, tau_t=0.1 0.1, ref_t=300 300, pcoupl=Parrinello-Rahman, pcoupltype=isotropic, tau_p=2.0, ref_p=1.0, compressibility=4.5e-5, pbc=xyz, DispCorr=EnerPres, gen_vel=no
